# Antioxidant Therapeutics in Parkinson’s Disease: Current Challenges and Opportunities

**DOI:** 10.3390/antiox10030453

**Published:** 2021-03-15

**Authors:** Ana Patricia Duarte-Jurado, Yareth Gopar-Cuevas, Odila Saucedo-Cardenas, Maria de Jesus Loera-Arias, Roberto Montes-de-Oca-Luna, Aracely Garcia-Garcia, Humberto Rodriguez-Rocha

**Affiliations:** 1 Departamento de Histología, Facultad de Medicina, Universidad Autónoma de Nuevo León, Monterrey 64460, Mexico; ana.duartejrd@uanl.edu.mx (A.P.D.-J.); yareth.goparcu@uanl.edu.mx (Y.G.-C.); odila.saucedocr@uanl.edu.mx (O.S.-C.); MDJESUS.LOERAARS@uanl.edu.mx (M.d.J.L.-A.); ROBERTO.MONTESDEOCALN@uanl.edu.mx (R.M.-d.-O.-L.); 2 Centro de Investigación Biomédica del Noreste, Departamento de Genética Molecular, Instituto Mexicano del Seguro Social, Delegación Nuevo León 40080, Mexico

**Keywords:** Parkinson’s disease, oxidative stress, antioxidants, clinical trials

## Abstract

Oxidative stress is considered one of the pathological mechanisms that cause Parkinson’s disease (PD), which has led to the investigation of several antioxidants molecules as a potential therapeutic treatment against the disease. Although preclinical studies have demonstrated the efficacy of these compounds to maintain neuronal survival and activity in PD models, these results have not been reflected in clinical trials, antioxidants have not been able to act as disease modifiers in terms of clinical symptoms. Translational medicine currently faces the challenge of redesigning clinical trials to standardize criteria when testing molecules to reduce responses’ variability. Herein, we discuss current challenges and opportunities regarding several non-enzymatic antioxidants’ therapeutic molecules for PD patients’ potential treatment.

## 1. Introduction

Parkinson’s disease (PD) is a progressive, chronic, and degenerative neurological disorder. It manifests as a movement disorder, tremor, bradykinesia, and rigidity. PD is the second most common neurodegenerative disease after Alzheimer’s disease [1,2]. The estimated prevalence in industrialized countries is 0.3% in the general population, and older than 60 years is around 1% [1]. The incidence seems to increase with age, reaching 0.3 patients for every 1000 people between 55 and 65, and 4.4 patients for every 1000 people older than 85 years [3].

The main pathological characteristic of PD is the progressive degeneration of dopaminergic neurons [4]. Dopaminergic neurons are susceptible to oxidative damage due to dopamine’s inherent metabolism, which is oxidized and generates reactive oxygen species (ROS), leading to cellular oxidative stress, where ROS have different macromolecular targets [5]. Furthermore, oxidative stress and neuroinflammation exert a synergistic effect on PD pathogenesis [6]. In situ or peripheral neuroinflammation, including glial cell activity [7,8], and pro-inflammatory cytokines [9], has been observed in post-mortem brain samples from PD patients, which might contribute to the cascade of events that lead to neurodegeneration. Once activated, glial cells are capable of regulating various enzymatic systems, such as NADPH oxidase, inducible nitric oxide synthase (iNOS), and myeloperoxidase [10], with the subsequent production of cytotoxic factors like superoxide anion (O_2_^•−^) and nitric oxide (NO^•^).

Substantial evidence suggests that oxidative stress is involved in the etiology and pathogenesis of neurodegenerative disorders. The brain is rich in polyunsaturated fatty acids. Therefore, it is highly susceptible to lipid peroxidation, which is a complex process that involves the interaction of fatty acids with free radicals generating reactive electrophilic aldehydes. Lipid peroxidation occurs in many neurodegenerative diseases [11]. Evidence of oxidative stress in these diseases is further supported by increased oxidation of DNA and RNA as well as oxidative protein damage in some brain areas [12]. In addition to lipids, DNA, and RNA, proteins are the main targets of oxidative stress, and their oxidated forms and byproducts are potential biomarkers in neurodegenerative diseases. Increased lipid peroxidation (evidenced by decreased polyunsaturated fatty acids and increased malondialdehyde and 4-hydroxy-2-nonenal) elevated 8-hydroxyguanosine (8OHG) as an indicator of nucleic acid oxidation, and high protein carbonylation and nitration (3-nitrotyrosine) have been detected in PD patients [13,14,15,16]. The destruction of some cellular components induces a diversity of responses through the generation of secondary reactive species (RS) and ultimately leads to cell death by apoptosis or necrosis [17,18,19].

The most conclusive association between PD and oxidative stress is supported by post-mortem studies of PD patients’ brains, where increased oxidative activity and macromolecules damage were found in dopaminergic neurons [20,21].

Antioxidants are enzymes, molecules, ions, or even very stable radicals capable of delaying or preventing another molecule’s oxidation. According to their activity, antioxidants can be classified into enzymatic and non-enzymatic. Enzymatic antioxidants break down and remove free radicals in a multistep process accompanied by cofactors, while non-enzymatic antioxidants interrupt free radical chain reactions [22]. Although molecules known as non-enzymatic antioxidants have shown neuroprotective effects in PD experimental models, they have failed to reproduce this protection in clinical trials. This review will discuss the current challenges and opportunities regarding several non-enzymatic antioxidants and their potential as a therapeutic treatment for PD patients.

## 2. Oxidative Stress and Antioxidant Defense

**Figure 1:**
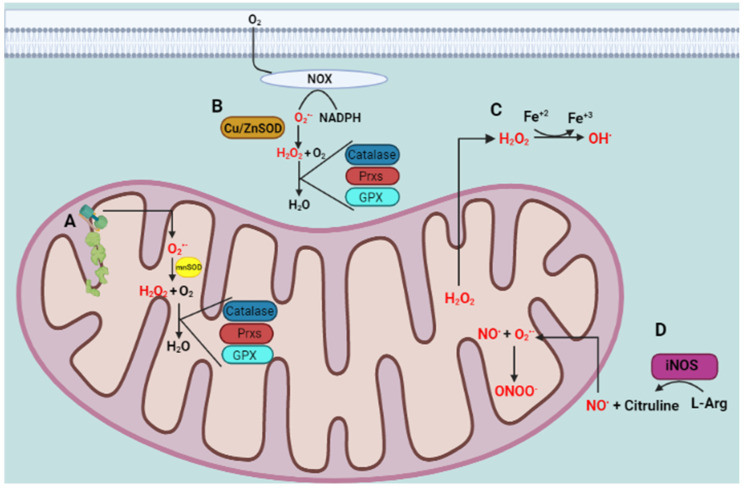
ROS and RNS generation. (**A**) The mitochondria are the main source of free radicals within the cell. The electron leakage in the electron transport chain (ETC) generates superoxide anion (O_2_^•−^) in the mitochondrial matrix, where it is scavenged by MnSOD producing H_2_O_2_ that, in turn, is detoxified by catalase, Prx, and GPx. (**B**) Another source of O_2_^•−^ is the NOX enzyme family, which uses NADPH to reduce molecular oxygen to produce O_2_^•−^. The O_2_^•−^ cannot cross membranes, but H_2_O_2_ easily diffuses through them. (**C**) If H_2_O_2_ concentration overcomes the enzymatic antioxidants activity, it reacts with transition metal ions such as iron, mediating the OH^•^ formation by the Fenton’s reaction. (**D**) Nitric oxide synthases (NOS) catalyze the L-arginine oxidation producing L-citrulline and NO^•^_._ The latter can diffuse through membranes and react with mitochondrial O_2_^•−^ producing peroxynitrite (ONOO^−^), which diffuses to different cellular compartments causing RNS-mediated damage (figure created in BioRender.com accessed on 29 January 2021).

**Figure 2:**
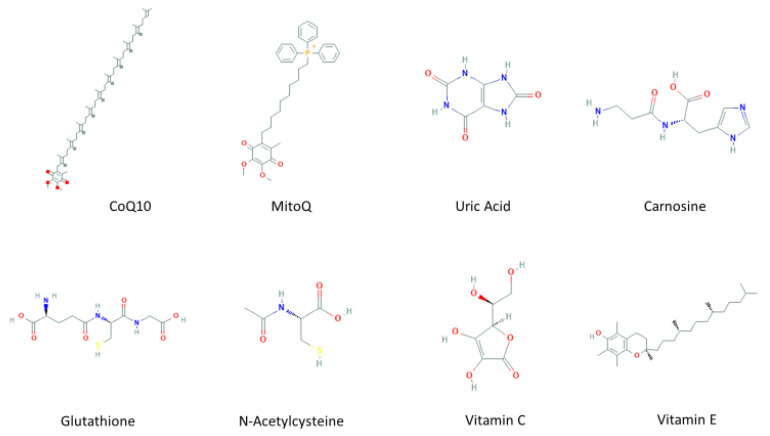
Molecular structures of non-enzymatic antioxidants.

An imbalance of RS production and reactive intermediates detoxification generates oxidative stress. Oxidative stress affects cellular function by targeting nucleic acids, lipids, and proteins, all of which are constituents of cell organelles [23]. RS comprises not only ROS, and reactive nitrogen species (RNS), but also reactive sulfur species (RSN), reactive carbonyl species (RCS), reactive halogen species (RHS), reactive selenium species (RSeS), and reactive nucleophilic species [24], generated by biological processes within the cell in normal and stressful conditions. Biomolecules are highly susceptible to oxidation by one or more ROS and RNS present in the environment, generated as by-products of normal metabolism, or during exposure to X, λ, or UV irradiation. In some cases, ROS are used against infectious pathogens or signaling pathways [25]. RS have a double and opposed function on cell fate. Under physiological conditions, a moderate RS increase plays an essential role in promoting cell proliferation and survival. However, when RS exceeds baseline levels, surpassing the cells’ antioxidant capacity, cell metabolic processes are affected. Usually, antioxidant defenses counteract oxidative stress and antioxidants along with the turnover of oxidated macromolecules and organelles, leading to cell survival. If oxidative stress persists, it can trigger cell death, including neuronal cell death or permanent cell damage producing cellular transformation [26]. ROS include many species, such as O_2_^•−^, hydroxyl radical (OH^•^), and hydrogen peroxide (H_2_O_2_), while RNS comprise NO^•^ and peroxynitrite (ONOO^−^). The primary source of ROS production within the cell are mitochondria (Figure 1). There, O_2_^•−^ is generated by electron leakage in the electron transport chain (ETC) and released to the mitochondrial matrix [27]. Another source is the nicotinamide adenine dinucleotide phosphate (NADPH) oxidase family (NOX enzymes), which uses NADPH to reduce molecular oxygen to produce O_2_^•−^ and release it to the cytoplasmic and extracellular compartments [28]. Usually, O_2_^•−^ is enzymatically dismuted to H_2_O_2_ by the superoxide dismutases MnSOD in the mitochondrial matrix, by Cu/ZnSOD in the IMS or cytosol [29], or EcSOD in the extracellular space [30]. Subsequently, H_2_O_2_ is detoxified in the cytoplasm and mitochondrial matrix by catalase [31], peroxiredoxins (Prx) [32], or the glutathione (GSH) peroxidase system (GPx) [33]. Although O_2_^•−^ cannot cross biological membranes [34], H_2_O_2_ freely diffuses through membranes [35] and reacts with metal ions mediating the highly reactive OH^•^ formation by the Fenton’s reaction [36]. The formation of NO^•^ is induced by NOS enzyme [37]. The reaction between NO^•^ and O_2_^•−^ is faster (0.7–1.9 × 10^10^ M^−1s−1^) than that with SOD (1–2 × 10^9^ M^−1^s^−1^). Therefore, NO^•^ overcomes the SOD reaction for O_2_^•−^ [38]. Consequently, every collision between NO^•^ and O_2_^•−^ results in the formation of ONOO^−^. The small hydrophobic NO^•^ molecule freely diffuses across membranes. Therefore, it is feasible that ONOO^−^ forms close to the O_2_^•−^ generation sites with the incoming NO^•^ molecules produced at a farther distance [39]. At physiological pH, ONOO^−^ crosses membranes and affects surrounding cells, reacting with thiol groups and transition metals. When ONOOH undergoes decomposition, it generates one-electron oxidants OH^•^ and (nitrogen dioxide) NO_2_^•^ [40]. In addition, the antioxidant scavenging system includes several non-enzymatic antioxidants (Figure 2), which are easy to obtain and modify the administration dose, including mitochondria-targeted antioxidant (MitoQ) [41], coenzyme Q10 [42], and carnosine [43], which are targeted to the mitochondria, the main source of free radicals; some endogenous antioxidants precursors like inosine, which is metabolized into uric acid (UA) [44], and N-acetylcysteine (NAC) [45], a glutathione (GSH) precursor; GSH itself [46], known as “the master antioxidant”; and vitamins C and E [47], whose effects on PD, from experimental to clinical, have been explored and will be discussed below.

## 3. Mitochondria-Targeted Antioxidants Therapeutic Effect on PD

### 3.1. Coenzyme Q10

Coenzyme Q10 (CoQ10) is a parabenzoquinone (also known as ubiquinone) ubiquitous in virtually all cells; it is a crucial component of the oxidative phosphorylation process in the mitochondria but is not just an agent for energy transduction. It is also well located close to the membranes unsaturated lipids, acting as a primary scavenger of free radicals to avoid lipid peroxidation [42], protecting biological membranes and DNA from oxidative damage [48].

The chemical structure of CoQ10 is very similar to vitamins; however, it is not considered one of them because it is the only lipid-soluble antioxidant that can be synthesized de novo by animal cells [49].

Ubiquinone endogenous levels depend on the production and consumption rates within the organism. The dietary intake of CoQ10 is minimal with daily contributions of around 3–5 mg [50], which explains why supplementation with CoQ10 has been recommended in cases of deficiency. Despite this, oral administration of CoQ10 has a poor absorption efficiency due to its hydrophobic nature. Therefore, various formulations have been created to improve its bioavailability [51]. Normal plasma CoQ10 levels are 0.5 to 1.5 μg/mL. Solubilized formulations administration increases the CoQ10 plasmatic concentration from doses of 100 mg [52], reaching levels of 3.25 μg/mL [53]. However, these concentrations need to be higher to promote its uptake by peripheral tissues and even cross the blood–brain barrier [54]. The highest CoQ10 plasmatic concentration is reached approximately 6 h after administration, and once no CoQ10 is free in circulation, it is mainly carried by LDL and VLDL lipoproteins. CoQ10′s half-life is 33 h, and it takes 1–2 weeks to reach a pharmacological steady-state [55]. Moreover, CoQ10 chronic oral administration increases mitochondrial CoQ10 concentration in the cerebral cortex attenuating striatal lesions and increasing life span in mice models [56].

Ubiquinone endogenous levels depend on the production and consumption rates within the organism. Ubiquinone levels are altered in some diseases associated with oxidative stress, including PD [57,58,59,60].

A significant CoQ10 concentration reduction was found in the cortex of PD post-mortem brain samples [61], while patients’ total CoQ10 plasma levels were decreased, in addition to an increased percentage of the oxidized form of ubiquinone, compared to healthy subjects [62]. More specifically, mitochondrial CoQ10 levels in PD patients’ platelets were lower than in age- and sex-matched control [63].

The effectiveness of CoQ10 as a protector against cell toxicity has been demonstrated in in vitro PD models. When striatal sections of adult mice were cultivated and treated with MPP^+^, the co-incubation with CoQ10 achieved a significantly reduced dopaminergic cell loss [64]. In addition, a CoQ10 pre-treatment reduced rotenone-induced cell death in human neuroblastoma cells [65]. These beneficial effects were reproducible in more complex experimental models. In African green monkeys, oral supplementation of 15 to 22 mg/kg per day for ten days before MPTP administration attenuated the loss of nigral dopaminergic neurons [66].

Clinical trials for CoQ10 in PD patients began more than two decades ago, initially with short studies and a few patients, finding that oral doses of 200 mg daily for three months were safe. However, no beneficial effect was obtained [67,68] (Table 1). After that, a more extensive study was performed with early PD-diagnosed patients, who were treated with doses up to 1200 mg per day, showing that CoQ10 was safe and well-tolerated and delayed the progressive function deterioration [69]. From these studies, doses and number of patients have been modified; however, the effects of preclinical trials have not been reproduced [70,71,72,73].

### 3.2. Mitochondria-Targeted Antioxidant (MitoQ)

An orally available derivative of mitochondrial-targeted coenzyme Q10 is a therapeutic compound termed Mitoquinone (MitoQ), which is an ubiquinone synthesized from the union of its oxidized (mitoquinone) and reduced (mitoquinol) form and a covalent bond to a lipophilic thriphenylphosphonium cation through an aliphatic carbon chain [41].

The MitoQ oral formulations have shown suitable pharmacokinetics behavior; doses of 1 mg/kg reach a maximum plasma concentration of 33.15 ng/mL after one hour of administration [74]. MitoQ is rapidly cleared from plasma and accumulates in the heart, skeletal muscle, liver, and brain; its accumulation came to a steady-state after 7 to 10 days of administration [75].

MitoQ quickly crosses the blood–brain barrier and cell membranes and concentrates on mitochondria because of its high membrane potential across the inner mitochondrial membrane [76]. Once inside, almost all the accumulated MitoQ is adsorbed to the inner membrane’s matrix surface, where it is reduced to the active antioxidant ubiquinol by complex II in the respiratory chain. MitoQ scavenges peroxyl radicals (ROO^•^), ONOO^−^ and O_2_^•−^ and protects mitochondria against lipid peroxidation. It is also a poor substrate for complex I and has no reactivity with complex III, so MitoQ remains in its reduced form ubiquinol, being more efficient than CoQ10 [77].

MitoQ proved to be protective in cellular and animal models of diseases related to oxidative stress. In a cellular PD model, MitoQ markedly inhibited 6-OHDA-induced mitochondrial fragmentation [78]. To support these findings, MitoQ treatment achieved an attenuation of both the loss of tyrosine hydroxylase (TH) and mitochondrial respiratory chain dysfunction induced by MPP^+^ in dopaminergic cells. In addition, the same effects were obtained from the PD animal model [79].

Despite these remarkable results, clinical interventions have not achieved the same impact. To determine whether MitoQ slows down or stops PD progression, different doses were tested in newly diagnosed PD patients in a double-blind clinical trial (Table 1). However, no difference was found between MitoQ treatments and the placebo group [80].

### 3.3. Carnosine

Carnosine (β-alanyine-l-histidine) is an endogenous dipeptide of excitable tissues (skeletal muscle, heart, and brain); it is highly hydrophilic, penetrates the blood–brain barrier easily, and has significant antioxidant properties. Carnosine is electrochemically active as a reducing agent; it shows peroxyl radical-trapping activity; inhibits deoxyguanosine oxidative hydroxylation induced by copper ions; and acts as a metal ion chelator, quenching singlet oxygen and binding hydroperoxides [43].

Carnosine concentration in the human brain is currently lacking in the literature. Homocarnosine (γ-aminobutyryl-l-histidine) is a novel alternative imidazole peptide with structural similarity to carnosine. Homocarnosine concentration in the human brain is quite high (0.4–1.0 µM). Both carnosine and homocarnosine are synthesized by carnosine synthase and degraded by carnosinase [81]

Carnosine restored intracellular ATP depleted by a 6-OHDA toxic effect in vitro and significantly suppressed the induction of stress-related genes activated by 6-OHDA [82]. An animal model with accelerated senescence under carnosine diet administration showed an increase in the average lifespan. This effect was accompanied by a decrease in mitochondrial MAO and Na/K-ATPase activity in synaptosome [83]. On the other hand, intranasal carnosine administration in a transgenic αSyn mice PD model decreased αSyn deposition in the olfactory epithelium [84].

In addition, a pilot study where PD patients with trembling-rigid manifestations received basic therapy for the disease (Levodopa and dopamine receptors agonist) and daily dietary supplementation with carnosine via oral showed increased efficiency of PD basic therapy, with neurological symptom improvement and pronounced SOD restoration, which is suppressed in PD [85]. Even with these promising results, carnosine’s efficacy has not currently been reassessed, either alone or in combination with the basic disease therapy or other antioxidants.

Although mitochondria as a target for antioxidants seems ideal because they are the main compartment where oxidative stress is produced, targeting oxidative molecules only in this subcellular compartment has been insufficient in patient’s therapy.

**Table 1:** Summary of mitochondrial antioxidants effect on PD clinical trials.

Antioxidant	Study Design	Clinical Trial	Subjects (n)	Primary Outcomes	Reference
Coenzyme Q10	Pilot study	Not applicable	10	Safe and well-tolerated No significant effect on the clinical ratings	[67]
	Pilot Study	Not applicable	15	Safe and well-tolerated No significant effect on the clinical ratings (UPDRS)	[68]
	Multicenter, randomized, parallel-group, placebo-controlled, double-blind, dosage-ranging	Phase II	80	Safe and well-tolerated at dosages of up to 1200 mg/d ↓ Disability developed, and the benefit was most significant in subjects receiving the highest dosage Slow the progressive deterioration of function in PD	[69]
	Randomized, placebo-controlled, double-blind clinical trial	Phase III	397 + 201	safe and well-tolerated No evidence of clinical benefit	[70]
	Randomized, double-blind, placebo-controlled, parallel-group pilot trials	Phase II	28 + 20	↓ UPDRS score	[71]
	Meta-Analysis	Phase III		Does not slow functional decline or provide any symptomatic benefits	[72]
	Double-blind, randomized, and placebo-controlled	Phase II	Total predict number: 84	This study focuses on genetically stratified subgroups of Parkinson’s disease patients (PD) with enrichment of risk variants in mitochondrial genes, who might benefit from treatment with the “mitochondrial enhancer” coenzyme Q10	[73]
MitoQ	Double-blind, placebo-controlled study	Phase II	128	Did not slow the progression of PD	[80]
Carnosine	Pilot comparative clinical trial	Phase I	36 + 20	↑ The efficiency of PD patients’ primary therapy. ↓ UPDRS score Restoration of SOD	[85]

## 4. Inosine Therapeutic Effect on PD

Inosine, also known as hypoxanthosine or panholic-L, belongs to the organic compounds known as purine nucleosides. It is an intermediate in the degradation of purines and purine nucleosides to UA and purine salvage pathways.

Inosine is a urate precursor, which has an antioxidant effect in vitro [44] and in vivo [86]. UA represents about 60% of total plasma antioxidant capacity [87]; it scavenges singlet oxygen (^1^O_2_), OH^•^, H_2_O_2_, and ONOO^−^ [88]. However, its effect is not limited to eliminating free radicals. UA-mediated neuroprotection has been improved by high K^+^-induced depolarization through a mechanism involving Ca^2+^ increasing and extracellular signal-regulated kinase 1/2 (ERK1/2) activation, which is involved in neuronal survival [89]. UA also interacts and stabilizes other antioxidant systems, including SOD [90]. UA has metal-complexing properties, too; it chelates iron by forming stable complexes with Fe^3+^ and blocking iron-dependent oxidation reactions [91].

Despite being the most abundant antioxidant in plasma, its concentration is ten times lower in cerebrospinal fluid (CSF). Its distribution is caudo-rostral gradient, at the highest concentration in the lumbar region and 50 times less at the brain stem level [92].

UA’s decreased concentration has been related to a higher susceptibility to oxidative damage [93] and a high-risk prediction for developing PD [94]. Interestingly, PD patients with higher UA plasma levels had a slow disease progression [95].

In neuron-like cells (PC12 cells), UA prevented oxidative damage induced by 6-OHDA by decreasing the levels of the lipid peroxidation biomarker malondialdehyde (MDA) as well as consumption of other antioxidants such as GSH and SOD [96]. This effect was also observed in a PD animal model induced by 6-OHDA, where GSH depletion, SOD activity decrease, and dopaminergic neuronal damage resulting from the 6-OHDA injury were reversed by intraperitoneal treatment with UA [86].

The previous evidence led to clinical trials to assess inosine’s effect on neurodegenerative diseases. Initially, clinical studies showed that, although inosine can increase plasma antioxidants, there is a lack of apparent inosine effect on CSF (Table 2). This may suggest that inosine’s antioxidant actions do not extend to the central nervous system, or at least to its CSF compartment [97]. Later, in the SURE-PD trial, it was observed that inosine produced more significant increases in serum and CSF urate in women compared to men [98]. Currently, a multicenter, randomized, double-blind, placebo-controlled phase 3 study is being completed, where 298 subjects were enrolled. Its main objective is to determine the urate precursor inosine’s early PD capability as a disease modifier.

Despite being widely studied as a biomarker and a risk predictor of PD, the specific mechanism of urate and its interaction with other antioxidants systems is not clearly defined, which is essential to achieve the preclinical effects when administered to patients.

**Table 2:** Summary of inosine antioxidant effect on PD clinical trials.

Antioxidant	Study Design	Clinical Trial	Subjects (n)	Primary Outcomes	Reference
Inosine	Prospective cohort study	NA	4695	↑ serum levels of UA ↓ risk of PD	[94]
	Double-blind randomized	NA	800	↑ serum and CSF UA ↓ clinical decline in PD	[95]
	Randomized, double-blind, placebo-controlled, dose-ranging	Phase II	75	↑serum and CSF urate levels in early PD	[98]
	Randomized, double-blind, placebo-controlled	Phase II	75	Dose-dependent and persistent elevation of plasma antioxidant capacity form oral inosine	[97]
	Placebo-controlled double-blind dose-ranging	Phase II b	75	↑ serum and CSF urate in women compared to men	[98]

## 5. Cysteine-Based Antioxidants Therapeutic Effect on PD

### 5.1. Glutathione

GSH is a tripeptide (cysteine, glycine, and glutamic acid) found in high concentrations in most cells’ mitochondrial and cytoplasmatic compartments. It is synthesized in the cytoplasm by the sequential addition of cysteine to glutamic acid, followed by glycine addition. GSH functions as an antioxidant, a free radical scavenger, and a detoxifying agent; it is a GSH peroxidase cofactor, acts as a substrate for GSH S-transferase, and maintains exogenous antioxidants, such as vitamins C and E, in their reduced (active) forms [99,100,101]. The cysteine’s sulfhydryl group is essential for GSH function as it participates in the reduction, oxidation, and conjugation reactions [102]. This antioxidant exists in two states: reduced (GSH) and oxidized (GSSG); in the reduced state, the cysteine’s thiol group can donate an electron to unstable molecules such as ROS; by donating this electron, the GSH oxidizes and reacts with another GSH to form GSH disulfide (GSSG). Therefore, the ratio of GSH/GSSG determines the cells’ redox status [103].

One of the biochemical alterations that had been detected in post-mortem brains of PD patients is the selective GSH reduction in the substantia nigra. In addition, the GSH/GSSG ratio was altered in favor of the oxidized form [104]. A similar pattern of GSH depletion has been found in incidental Lewy body diseases, which is considered a presymptomatic form of PD [105]. Additionally, under significant oxidative stress conditions as normal aging, decreased GSH leads to dopaminergic cell loss [106].

The reduction of GSH (by an irreversible inhibitor of g-glutamylcysteine synthase) potentiated the glutamate and NO cytotoxicity in rat mesencephalic dopaminergic cells [107]. Similarly, in mice, GSH depletion increased MPTP/MPP^+^ toxic effects, suggesting that an insufficient GSH pool may contribute to dopaminergic cell death in PD [108].

In vivo and in vitro experiments have shown that replenishing intracellular GSH levels can prevent oxidative damage and maintain mitochondrial function in dopaminergic cells [109], suggesting that restoring GSH levels in PD patients’ brains is a promising strategy to modify the disease’s progression.

Unfortunately, these effects have not been reproduced in clinical trials. Oral GSH supplementation with 500 mg twice daily for four weeks showed no changes in oxidative stress biomarkers, including GSH itself [110]. However, administering 1000 mg of GSH for six months increased plasma GSH levels by 35% [111]. Despite this, the restoration of the GSH pool remains challenging because of its physicochemical properties. Its high degradation rate in the gastrointestinal tract by γ-glutamyltransferase [112] reduces the probability to reach significant blood levels, and therefore enough concentration in CSF. 

To avoid GSH first-pass loss in the digestive tract, it was administered intravenously to PD patients in two separate clinical trials. The first one showed symptomatic efficacy with two daily GSH doses of 600 mg for 30 days in a small group of newly diagnosed and untreated PD patients (Table 3) [113]. In the second study, PD patients whose motor symptoms were not well controlled with their medication regimen were subjected to GSH doses of 1400 mg three times a week for four weeks [114], finding that GSH was well-tolerated and safe and suggesting the possibility of a slight symptomatic effect. However, both studies’ symptomatic improvements should be verified in a more extensive study with more subjects to detect significant differences.

Alternative routes of GSH administration have been tested, including noninvasive intranasal delivery. A safety survey showed that 62% of the subjects experienced health benefits from GSH, while 12% reported adverse effects [115]. A phase I/IIa clinical study in PD patients showed that intranasal GSH was safe and well-tolerable over a three-month intervention period [116]. Phase IIb of the study reported predicted improvements in PD total and motor scores, although they were not significantly different from the placebo group. In addition, one participant developed cardiomyopathy [117], which may delay future studies.

### 5.2. N-Acetylcysteine (NAC)

Acetylcysteine is a synthetic N-acetyl derivative of the endogenous amino acid l-cysteine, a GSH precursor (PubChem). NAC has been used for more than 50 years in the clinic to replenish hepatic GSH after acetaminophen overdose [118], as a mucolytic in lung diseases [119], and as a disease modifier in infectious diseases [120]. NAC’s antioxidant activity is attributed to its rapid reaction with OH^•^, NO_2_^•^, CO_3,_ and thiyl radicals, as well as to the detoxification of semiquinones, hypochlorous acid (HOCl), nitroxyl (HNO), and heavy metals [45].

NAC is a membrane-permeable cysteine precursor. Since neurons cannot take up GSH itself, NAC is a potential cysteine source necessary to stimulate enzymes involved in the brain’s GSH generation, representing a promising therapeutic molecule in treating PD. NAC’s beneficial effect has been shown in human cortical cells exposed to toxic agents and inducers of neuronal death, where mitochondrial homeostasis, and consequently apoptosis inhibition, was observed [121]. Additionally, several beneficial effects have been observed in vivo, including increased endogenous GSH levels in cortical synaptosome, which are sites of increased oxidative stress in neurodegeneration [122]; increased Complex I activity in aged mice synaptic mitochondria [123]; improved cytochrome C oxidase in young and old mice [124]; and even GSH oral administration protected from the loss of dopaminergic cells in α-synuclein (αSyn) overexpressing mice [125].

However, NAC has a low oral bioavailability estimated at 6–10% due to extensive first-pass metabolism [126]. After an hour, a single oral dose of NAC 200 to 400 mg reaches the peak plasma concentration of 0.35 to 4 mg/L and has a half-life of 6.25 h after administration [126]. When administered intravenously, systemically, NAC binds to plasma proteins, and about 30% undergoes renal elimination resulting in a short half-life [45,126]. Importantly, increasing systemic doses of NAC to raise its transport across the brain-blood barrier may increase blood pressure [127].

Nevertheless, preclinical studies suggest NAC’s potential effectiveness as a PD modifier and have led to performing some clinical trials (Table 3). In a trial with Parkinson’s and Gaucher’s disease patients, brain GSH levels were quantified by magnetic resonance spectroscopy after a single intravenous infusion of 150 mg/kg NAC; although GSH was increased in all subjects, it returned to basal levels after 120 min [128]. A pilot study was performed to evaluate the safety, tolerability, and preliminary efficacy of NAC intravenous and oral administration; the data obtained suggested that NAC might be positively impacting dopamine function and potentially clinical symptoms [129]. Each subject was provided with 3000 mg NAC twice a day (6000 mg total) for four weeks in a prospective study. The results of this study showed that, although repeated oral doses of NAC increased the antioxidants (GSH/GSSG) peripherical levels, the indicators of oxidative damage (lipid peroxidation) remained unchanged, and the GSH brain levels did not increase significantly [130]. The most recent clinical trial recruited PD patients, who received a combined oral and intravenous NAC administration for three months. Afterward, patients were evaluated by SPECT imaging and UPRDS (Unified Parkinson’s Disease Rating Scale) score, which showed that NAC improved dopamine transporter (DAT) binding in PD patients, correlating with improvement symptoms [131].

**Table 3:** Summary of Glutathione and NAC antioxidants effect on PD clinical trials.

Antioxidant	Study Design	Clinical Trial	Subjects (n)	Primary Outcomes	References
Glutathione	Pilot evaluation	Phase I	9	GSH has symptomatic effects and possibly retards the progression of the disease	[113]
	Randomized, Double-Blind, Pilot Evaluation	Phase I	11 + 10	Safe and well-tolerated	[114]
	Randomized, double-blind	Phase I/IIa	15 + 15	These data support the safety and tolerability of intranasal GSH in this population Pharmacokinetic and dose-finding studies are warranted	[115,116]
	Double-blind placebo-controlled	Phase IIb	45	All cohorts improved over the intervention period, although neither treatment group was superior to the placebo	
N-Acetylcysteine	Interventional Open label	Phase I	6 + 2	↑GSH redox ratios ↑ Brain GSH	[128]
	Single-center	Phase I	12	Orally ↑ CSF NAC concentrations	[132]
	Randomized open-label	NA (preliminary)	65	↑ DAT binding in the caudate and putamen	[129]
	Randomized controlled trial	NA	65	↑ DAT binding in patients with PD ↓ UPDRS score	[131]

## 6. Vitamin Therapeutic Effect on PD

### 6.1. Vitamin C

Vitamin C (Vit C), also known as ascorbic acid, ascorbate, or L-ascorbate, is a natural water-soluble vitamin that plays significant roles as a free radical scavenger and as a cofactor of several enzymes reactions, including catecholamine synthesis [133]. Its effect as an antioxidant comes from its actions as a non-enzymatic reducer of O_2_^•−^, hydroxyl (HO^•^), alkoxyl (RO^•^), peroxyl (ROO^•^), and other radicals [134]. Vit C also reacts with the radical tocopheroxyl, which results from Vitamin E oxidation when it scavenges free radicals in lipid membranes, regenerating Vitamin E (Vit E) [135].

Unlike most molecular low-weight compounds, the absorption, distribution, and metabolism of Vit C are complex. Its uptake in tissues and distribution mainly occurs through the sodium-dependent vitamin C transporter family of proteins [136]. These transporters’ differential expression between tissues leads to nonlinear pharmacokinetics of Vit C under physiological conditions, and its distribution is highly compartmentalized [137]. Vit C distribution pattern has a wide range of concentrations in the different tissues ranging from 0.2 mM in muscle and heart to 10 mM in the adrenal glands and brain [138]. 

The brain is one of the organs with the highest Vit C concentration [139]. However, it has a variable distribution in different brain regions, and the motor circuit-related sites receive the least Vit C concentration [140]. The brain has specific transporters for its reduced (SVCT2, sodium-dependent vitamin C transporter-2) and oxidized form (GLUT family) [136].

Low plasma levels of exogenous antioxidants (including Vit C) have been reported in patients with neurodegenerative diseases such as PD [141]. A higher prevalence of PD with subclinical Vit C deficiencies has also been received [142]. The monitoring of Vit C levels in lymphocytes has been suggested as a potential biomarker of PD progression [143].

The above suggests that Vit C supplementation is a potential therapeutic adjuvant in PD treatment. Positive results in PD experimental models have supported this theory. The supplementation with Vit C had a neuroprotective effect against levodopa’s neurotoxicity in vitro [144]. Vit C high doses and long treatments induced neuroprotection but physiological side effects in a fly model [145]. Vit C supplementation in 6-OHDA-lesioned rats showed an increase in dopaminergic neurons [146].

However, when Vit C was tested in the clinical trials, its effects were not reproduced. Vit C’s dietary intakes were related to a decreased PD risk in two large cohort prospective studies; however, this effect was not substantial in a 4-year lag analysis [147,148] (Table 4). A pilot clinical trial showed that a high dosage of Vit C and α-tocopherol, which is the most prevalent form of Vit E, delayed the start of levodopa therapy up to 2.5 years in newly diagnosed PD patients [149]; Vit C intake also modified the levodopa pharmacological kinetics in PD patients, increasing its absorption and bioavailability [150]. However, Vit C did not significantly reduce the risk of developing PD [29].

### 6.2. Vitamin E

Vit E is the generic term for eight substances or trocochromanols, four tocopherols, four tocotrienols [123], and the major lipid-soluble antioxidant [151]. As this vitamin cannot be synthesized in the human body, it must be supplied by the diet. The antioxidant effects exerted by each Vit E isoform are complicated, and their mechanisms are still not well understood. However, researchers theorize that Vit E may protect key cell components by reducing free radicals and breaking lipid peroxidation chain reaction. Thus, cell membranes are protected by lipid repair and replacement [152].

As mentioned above, the antioxidant effects of Vit E and its mechanism of action are not well understood. Vit E absorption efficiency varies from 10 to 33% [153] and is affected by several factors including the food matrix, genetic factors, and metabolic fate, altering its bioavailability [154].

Preclinical studies of PD have yielded controversial results regarding Vit E’s usefulness as a disease modifier. Vit E deficiency increased MPTP toxicity in the substantia nigra, while partially protected from neurotransmitter and metabolite depletion induced by MPTP in the striatum [155]. However, systemic administration of Vit E did not protect mice against MPTP toxicity [156]. Even α-tocopherol treatment before MPTP administration was unable to attenuate dopamine depletion [157]. Other studies support the theory that high Vit E doses partially protect dopaminergic neurons against MPTP-mediated toxicity; however, this protective effect ended once the antioxidant administration was stopped [158].

The multicenter controlled clinical trial Deprenyl and Tocopherol Antioxidative Therapy of Parkinsonism (DATATOP) recruited early and untreated PD patients (Table 4), who received deprenyl (10 mg per day) and tocopherol (2000 UI per day). Deprenyl (monoamine oxidase inhibitor) delayed for almost nine months the development of disability requiring levodopa therapy. However, the slight motor performance improvement became slightly worse upon deprenyl withdrawal. Nevertheless, there was no beneficial effect of tocopherol or any interaction between tocopherol and deprenyl [159].

Vit C and α-tocopherol combined administration delayed the start of levodopa therapy by 2.5 years in newly diagnosed PD patients [149].

**Table 4:** Summary of vitamins antioxidant effects on PD clinical trials.

Antioxidant	Study Design	Clinical Trial	Subjects (n)	Primary Outcomes	Reference
Vitamin C	Prospective cohort study		76,890	Do not reduce the risk of PD	[147]
	Comparative study	Phase I	67	↑ Absorption and bioavailability of levodopa	[150]
	Randomized Double-blind Controlled Trial	Phase II	76	Extend the time necessary to start levodopa therapy to 2.5 years	[149]
Deprenyl and Tocopherol	Placebo-Controlled	Phase I	800	There was no beneficial effect of tocopherol or any interaction between tocopherol and deprenyl	[159]
	Double-blind, randomized clinical	Phase II	199 + 191	↓ UPDRS score	[160]
	Prospective cohort study		1032	↓ Parkinson’s disease risk (this result was not significant in a 4-y lag analysis)	[148]

## 7. Conclusions

Oxidative stress has long been considered one of the pathophysiological mechanisms involved in PD, which has led to the investigation of the antioxidant systems as a promising therapy more than two decades ago. A useful antioxidant must meet specific characteristics; it must be capable of interacting with biologically relevant oxidants and free radicals; its reaction by-products should be harmless; and, finally, it must reach a sufficiently high concentration in the tissue and cell compartments to ensure that its activity is quantitatively relevant.

**Figure 3:**
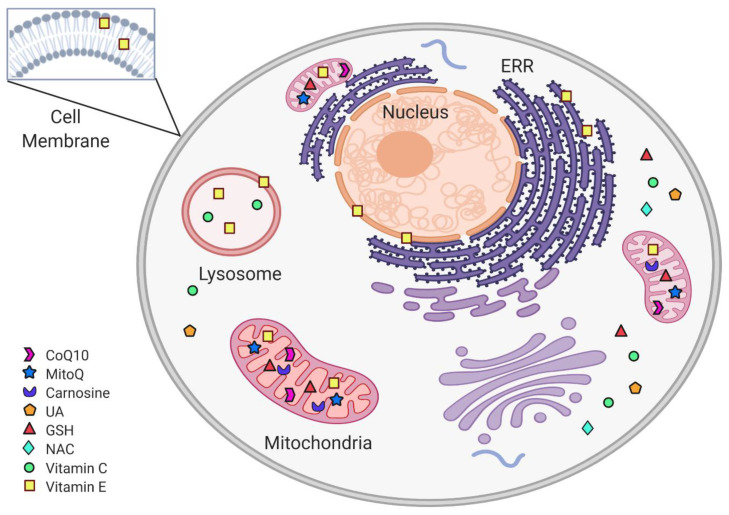
Subcellular non-enzymatic antioxidants targets. Within the cell, non-enzymatic antioxidants are distributed according to their subcellular target. The mitochondria are protected from oxidative stress by CoQ10, MitoQ, carnosine, and Vit C. In the cytoplasm, the antioxidant system includes GSH, NAC, and UA. Vit E is found in cytoplasmic, ERR, and mitochondrial membranes, as well as in lysosomes.

Various antioxidants’ administration has successfully maintained neuronal survival and preserved and improved the neurons’ antioxidant system’s activity in preclinical PD models. Unfortunately, when the experimental models are translated into clinical trials, these therapies have been less effective. However, the failed non-enzymatic antioxidants’ clinical trials in PD do not necessarily rule out the possibility of success in the future. What are the current challenges and opportunities? Despite the mechanistic evolutionary similarities between humans and rodents, which are the primary choice for research, lab rodents are syngeneic and are maintained under control conditions, while the heterogeneous human nature of PD complicates the outcome. In addition, the promising antioxidant effects observed in animal PD models cannot be translated into clinical trials because preclinical studies usually use young animals. Aged animal models are more suitable to analyze the antioxidant effect on PD. In addition, 25% of the cases diagnosed as PD are incorrect [161]. Since no objective molecular or biochemical tests exist for PD, its diagnosis is based on clinical criteria, mainly cardinal motor symptoms [162], which manifest when patients have lost about 60–80% of dopaminergic neurons [163]. Clinical trials usually include patients in advanced PD stages, with a considerable dopaminergic neuronal loss, and antioxidant therapy hardly made a significant difference. Until an accurate diagnosis method is available to discern between early, moderated, and advanced PD stages, antioxidant therapy’s real effectiveness will be determined. Current challenges for translational medicine include short antioxidant half-life, subcellular compartment-specific targeting delivery (Figure 3), suitable antioxidant bioavailability, high redox reaction rate, and specificity between antioxidants and oxidants. Antioxidant bioavailability is related to the antioxidant structure, interaction with other molecules, half-life, and delivery efficiency into the brain. Increasing the antioxidant dosage would not necessarily increase its tissue-specific concentration; it actually may produce adverse effects. To improve the antioxidant half-life, tissue-specific delivery, and bioavailability, antioxidants can be incorporated into a biocompatible substrate to surpass the administration limitations and increase its therapeutic effect.

Recently, antioxidant delivery and transportation systems were developed for specific subcellular targeting [164] by encapsulation into liposomes or linkage to nanoparticles including nanovesicles, solid lipid nanoparticles, nanostructured lipid carriers, nanoemulsions, and polymeric nanoparticles to make them more stable [165,166] or encapsulate them into liposomes. These are up-and-coming systems to be evaluated in preclinical and clinical studies to obtain the most significant antioxidants benefits, with the least toxicity and side effects.
